# Nitrates and Nitrites in Leafy Vegetables: The Influence of Culinary Processing on Concentration Levels and Possible Impact on Health

**DOI:** 10.3390/ijms26073018

**Published:** 2025-03-26

**Authors:** Sanja Luetic, Zlatka Knezovic, Katarina Jurcic, Marina Luetic Perasovic, Davorka Sutlovic

**Affiliations:** 1 Teaching Institute for Public Health, Split-Dalmatia County, 21000 Split, Croatia; sanja.luetic@nzjz-split.hr (S.L.); zlatka.knezovic@nzjz-split.hr (Z.K.); katarina.jurcic@nzjz-split.hr (K.J.); 2 Department of Health Studies, University of Split, 21000 Split, Croatia; 3 Institute of Emergency Medicine of Split-Dalmatia County, 21000 Split, Croatia; marina.luetic95@gmail.com; 4 Department of Applied Pharmacy, School of Medicine, University of Split, 21000 Split, Croatia

**Keywords:** nitrates, nitrites, leafy vegetables, culinary procedure, reduction factor

## Abstract

Vegetables, as an important source of vitamins and minerals, are highly recommended in a healthy diet. At the same time, vegetables can contain elevated amounts of nitrates and nitrites, which are the possible nitrosating agents responsible for the formation of carcinogenic nitrosamines. In young children, they can cause methemoglobinemia. Determining the level of nitrates and nitrites, as well as the possible reduction in their concentrations during culinary processing, is especially important for the diet of young children, who are introduced to leafy vegetables during the first year. For some types of vegetables that are often found in the diet, maximum permissible concentrations have not yet been established. Our goal was to estimate the reduction factors of nitrates and nitrites and suggest the best ways to properly prepare foods. For this purpose, samples of Swiss chard, spinach, and white cabbage were collected from the market to determine the nitrate and nitrite content. Vegetable samples were subjected to culinary preparations: soaking, cooking, and a combination of soaking and cooking. Quantitative and qualitative determination of nitrates and nitrites in vegetables was carried out on high-performance liquid chromatography (HPLC) equipped with a diode array detector (DAD). The obtained results showed that the highest nitrate concentrations were in Swiss chard samples, followed by spinach, and the lowest in white cabbage samples. The impact of culinary preparation was highest on spinach samples. Considering the average nitrate concentrations achieved after cooking or soaking and cooking, there was no risk of exceeding the ADI limit. However, the ADI values would be exceeded at the maximum nitrate concentrations.

## 1. Introduction

People with inherent or acquired genetic predispositions who are exposed to a range of environmental hazards and circumstances can develop cancer. One of the key factors in the possible development of gastrointestinal (GI) cancers is diet. Diets that are irregular, particularly those that include a lot of “red” meat—which is high in fat and protein—may raise the risk of gastrointestinal cancers. A high-fat diet causes the secretion of more bile acids, which the colonic microbiota transforms into secondary bile acids that are genotoxic—damaging DNA with reactive oxygen and nitrogen species [1]. Additionally, the microbial fermentation of undigested protein residues in the intestines produces inflammatory and toxic metabolites like phenols, indoles, and amines that can be precursors for the creation of carcinogenic N-nitroso compounds (NOCs) [1].

Dietary nitrates and nitrites can also act as precursors for the formation of NOCs [2]. After ingestion, nitrate is transported to the bloodstream. Most nitrate is eliminated through the urine, but 25% of nitrate is reabsorbed from plasma by the salivary glands and in oral cavities, approximately 5–7% of dietary nitrate is reduced to nitrites [3]. Depending on stomach conditions (pH, presence of certain bacterial species, type of consumed food) nitrites can become part of the N-nitrosamines formation cycle [4].

N-nitrosamines, especially N-nitrosodimethylamine (NDMA), have been proven to cause cancer in the stomach and intestines. Their direct contact with DNA, which leads to detrimental reactions like deamination and nitration, is the main cause of their carcinogenicity [5]. After being created, N-nitrosamines can enter target tissues and be broken down by cytochrome P450 (CYP) enzymes. During this metabolic activation, N-nitrosamines are transformed into extremely reactive electrophilic species that can covalently bond to DNA and cause the alkylation of DNA bases [6]. These adducts can induce DNA damage that can result in mutations during DNA replication, which can disturb normal cell processes and promote carcinogenesis [6,7]. Also, haemoglobin can react with nitrite in the blood causing a reduction in oxygen transmission capacity and possible development of methemoglobinemia, known as “blue baby syndrome” [8].

Due to the consumption of large amounts of nitrates and nitrites, the possibility of creating nitrosamines increases, which is associated with an increased risk of stomach and esophageal cancer [9]. However, there are studies that indicate a possible positive effect of dietary nitrates and nitrites. Namely, nitrite formed by nitrate reduction in the mouth can be a substrate from which reactive nitrogen intermediates are formed, with possible beneficial effects on maintaining immunity and physiological homeostasis in the body [10]. In addition, some studies have found a positive correlation between the consumption of nitrate-rich food and type 2 diabetes and gastrointestinal cancer [11] as well as positive effects on cardiovascular health, lowering blood pressure, and protecting against gastric injury and bleeding. Some researchers have pointed to the possible role of antioxidants present in vegetables (ascorbic acid, polyphenols, and carotenoids), which can reduce the formation of nitrosamines through the reduction of nitrites to nitrite oxide [12].

The European Agency for Food Safety (EFSA), in a report on the reevaluation of nitrite salts as food additives stated considering exposure to nitrites from all sources (food additives, natural presence, and contamination), found that acceptable daily intake (ADI) would be exceeded for infants, toddlers, and children at the mean and for all age groups at the highest exposure [13]. The EFSA also recommended the enforcement of comprehensive prospective studies on NDMA, nitrite and nitrate intake, and the risk of colon cancer (CRC). The decision was based on the IARC evaluation of the epidemiological studies on nitrate, nitrite, and cancer with the conclusion that from the evaluated studies, there was evidence for a positive association between preformed NDMA and increased risk of CRC or its subtypes [13,14].

Vegetables are an important part of a healthy Mediterranean diet that provides sufficient levels of essential vitamins, minerals, and dietary fiber [15,16]. The Mediterranean diet particularly recommends leafy vegetables such as Swiss chard, spinach, and cabbage due to their high nutritional value. However, these vegetables can accumulate high amounts of nitrates, either from natural sources in the environment or as a result of excessive artificial fertilizer use. Namely, in order to accelerate growth and boost vegetable yield, the usage of nitrogen fertilizers on farms is extensive [17]. The nitrogen absorbed by plants is converted to nitrate during plant growth. The concentration of nitrates and nitrites in vegetables is affected by the application rate as well as the amount of fertilizer [18,19]. Cultivation conditions, especially climatic ones, affect nitrate chemism, so growing in greenhouses or in geographical areas with less sunlight can lead to increased nitrate concentration due to reduced nitrate reductase activity [20]. Vegetables are the most important source of dietary nitrate, accounting for 50–70% of the overall intake [21]. Estimates are that the vegetarian population is more exposed to nitrite risk due to their higher consumption of vegetables with a high nitrate content [22]. Special concern should be addressed to infants and young children, who are more susceptible to nitrate-related health problems since leafy vegetables are introduced in their complementary feeding in the first age of life.

Cooking processes such as washing and boiling have been shown to affect the nitrate content of vegetables, leading to reduced concentrations. However, the extent of these reductions depends on the type of vegetable as well as the preparation method [23,24,25,26].

It should be emphasized that no residue limits for nitrates have been set for some types of leafy green vegetables, e.g., for Swiss chard, although several studies have shown significantly higher concentrations of nitrates in chard than in vegetables for which there are residue limits [27,28,29]. There are no residue limits set for nitrites for any type of vegetable [30].

Therefore, the aim of this study was to determine the concentrations of nitrite and nitrate in fresh vegetables as well as their remaining concentrations after different culinary processing. The focus of our research was on vegetables common in the infant and young children’s diet and for which there are no legal restrictions regarding nitrate concentration. By quantifying these changes, the study seeks to provide valuable insights into safe preparation practices and contribute to the development of dietary guidelines that promote a healthy diet while ensuring safety in terms of nitrate and nitrite intake, which is especially important for infants and toddlers.

## 2. Results

### 2.1. Nitrate Concentrations

**Figure 1:**
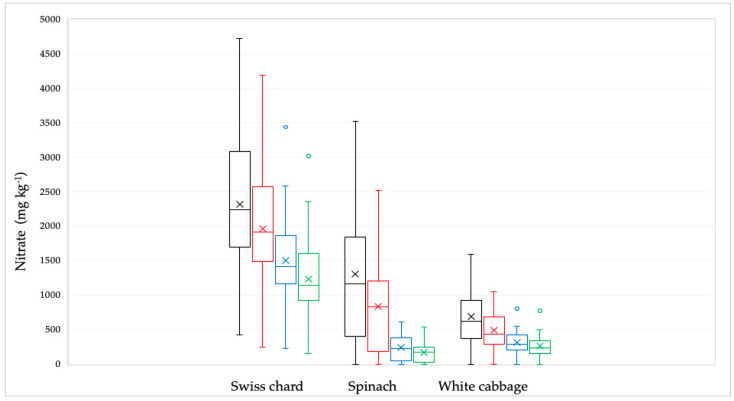
Distribution of the nitrate concentrations in Swiss chard, spinach, and white cabbage depending on the culinary processing: raw—black line; soaked in water—red line; boiled in water—blue line and combined, soaked and boiled—green line. The bars and the whiskers represent the minimum, maximum, mean (x), and median.

Of the 92 samples analyzed, the highest nitrate concentration was found in the Swiss chard sample (4723 mg kg^−1^) while the lowest concentration was determined in spinach and white cabbage samples (<Limit of Quantitation, LOQ). Observing all the samples, the lowest mean values were found in white cabbage, while the highest mean values were found in Swiss chard (Table 1, Figure 1).

**Figure 2:**
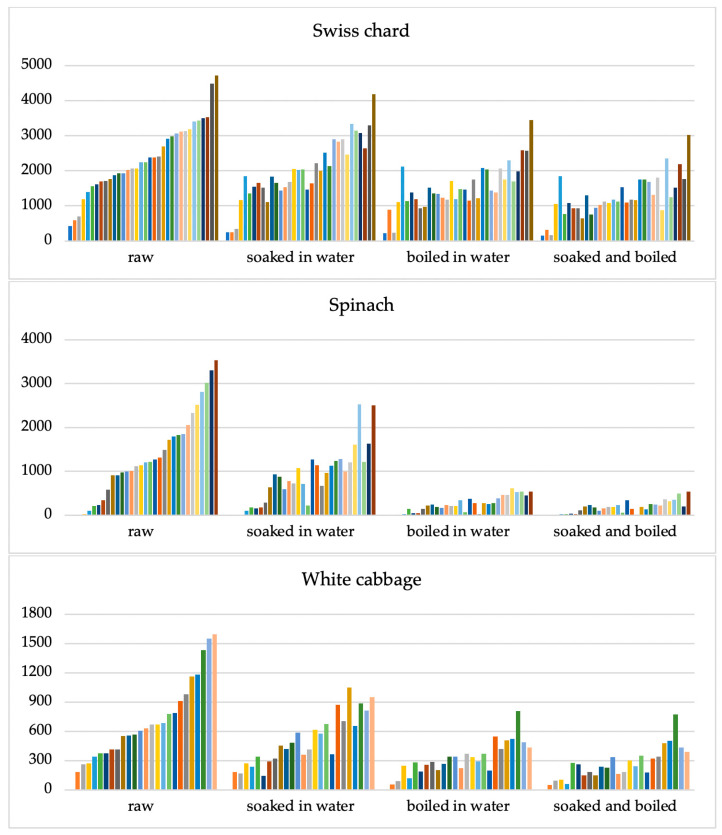
Clusters of nitrate concentrations in three types of vegetables: Swiss chard (*n* = 34), spinach (*n* = 32), and white cabbage (*n* = 26) in raw vegetables and according to different culinary processing methods: soaking for 15 min in deionized water, boiled for 10 min at 100 °C in deionized water, and combination of soaking and boiling processes. The Y-axis shows nitrate concentrations (mg kg^−1^). Each line of a different colour represents the result of an individual sample.

The nitrate concentration in the samples decreased significantly (*p* < 0.001) after being treated by soaking in water, boiling in water, and their combination The nitrate concentration clusters of all examined samples utilizing various culinary methods are displayed in Figure 2.

**Figure 3:**
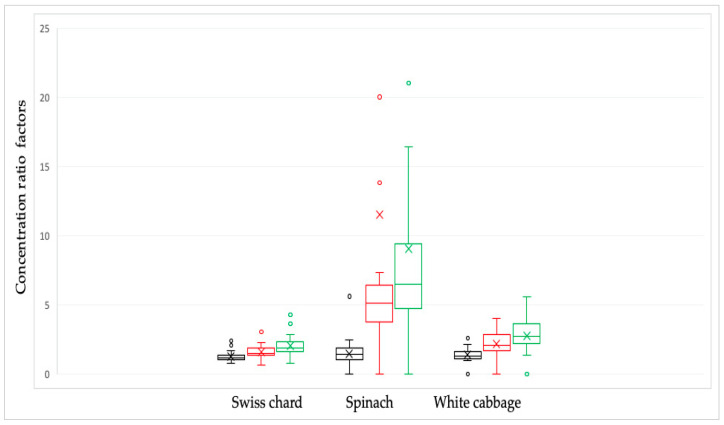
Nitrate concentration ratio factors in Swiss chard, spinach, and white cabbage depending on culinary processing: raw/soaked in water—black line; raw/cooked in water—red line and raw/combined, soaked and cooked—green line. The bars and the whiskers represent the minimum, maximum, mean (x), and median.

The combination of the soaking and cooking methods had the biggest effect on the nitrate content, whereas the vegetable soaking method had the least effect. Table 2 and Figure 3 display the conversion factor’s results.

The greatest impact on reducing nitrate concentration was observed for spinach samples (Figure 2).

Changes in culinary processing between soaking and boiling were determined using the Pearson correlation coefficient. The results showed that there was a statistically significant (*p* < 0.05) correlation in the ratios of nitrate concentrations with respect to the preparation methods. Correlation factors were moderately strong for spinach samples (R = 0.715), while a weaker correlation was observed in white cabbage samples (R = 0.415). For Swiss chard samples, there was a weak correlation (R = 0.231). A stronger correlation means that nitrate reduction was achieved equally by applying one of the two culinary processing, while a weak correlation factor points to differences in nitrate concentration with respect to different culinary processing.

### 2.2. Nitrite Concentrations

**Figure 4:**
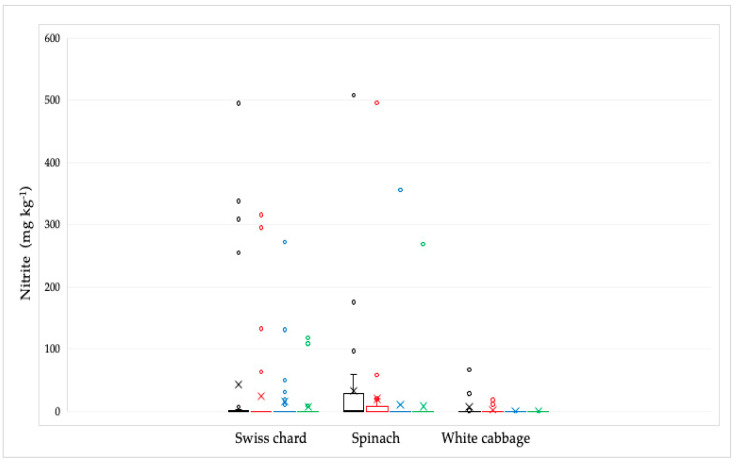
Distribution of the nitrite concentrations in Swiss chard, spinach, and white cabbage depending on the culinary processing: raw—black line; soaked in water—red line; boiled in water—blue line and combined, soaked and boiled—green line. The bars and the whiskers represent the minimum, maximum, mean (x), and median.

Out of 92 analyzed samples, the presence of nitrite was proven in 31 samples (Figure 4). The highest concentration was found in spinach samples, followed by Swiss chard, and the lowest in white cabbage samples. The highest nitrite concentration was found in the spinach sample (507.7 mg kg^−1^). The average values ± standard deviation (SD) in mg kg^−1^ were: 43.20 ± 119.70 for Swiss chard samples, 33.50 ± 93.80 for spinach samples, and 8.00 ± 20.20 for white cabbage samples. Their concentration was significantly reduced by culinary processing, especially by cooking, most notably in white cabbage and spinach samples, except in one spinach sample that had the highest concentration. In this sample, the nitrite concentration was reduced from the initial value after soaking from 507.7 to 495.8 and after cooking to 356 mg kg^−1^, while the combined culinary process reduced this value to 268.8 mg kg^−1^. In the Swiss chard samples, the concentration of nitrite was reduced by cooking, but not completely, probably because the initial concentrations were also very high in the raw sample. In general, in samples with nitrite concentrations less than 100 mg kg^−1^, all nitrites are mostly removed by culinary processing.

### 2.3. Calculation of Nitrate and Nitrite Intake After Consuming 100 G of Vegetables

**Figure 5:**
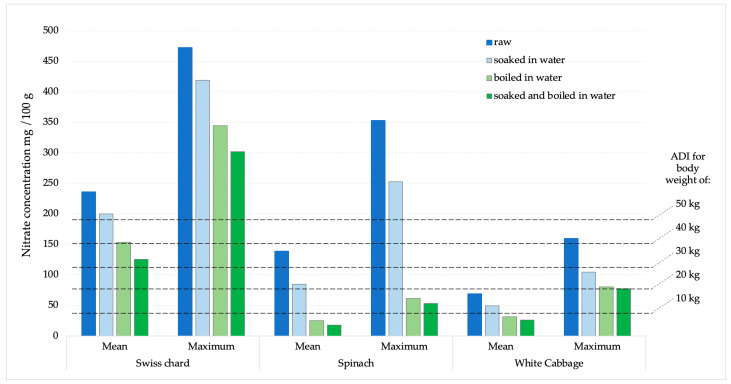
Presentation of mean and maximum nitrate values in analyzed products compared to ADI values for children of different body weights.

**Figure 6:**
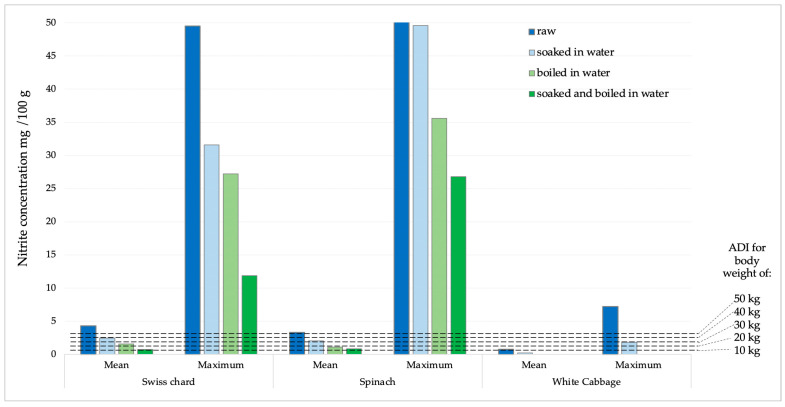
Presentation of mean and maximum nitrite values in analyzed products compared to ADI values for children of different body weights.

The potential daily intake of nitrates and nitrites was determined based on the consumption of 100 g of Swiss chard, spinach, or white cabbage. The calculations were made using the average and maximum concentrations obtained from the analysis of the sampled products (Figure 5 and Figure 6). According to the acceptable daily intake (ADI) for nitrate (0–3.7 mg kg^−1^ body weight, b.w.) and nitrite (0–0.07 mg kg^−1^ b.w.) [31], the achievement limits were assumed for people of different body weights (10–50 kg).

## 3. Discussion

The focus of the investigation was the nitrate concentrations in Swiss chard samples since the restriction on the maximum allowed nitrate levels does not include this vegetable. This type of vegetable is traditional in the menus of Mediterranean countries and is one of the first vegetables to be introduced into the diet of infants and toddlers. Additionally, we conducted an examination of nitrite residues following different culinary treatments because there is no residual restriction for nitrites in leafy greens. The highest nitrate concentration was recorded in raw Swiss chard samples, with a maximum value in one sample of 4723 mg kg^−1^. After Swiss chard, the highest nitrate values were found in raw spinach samples with 3528.70 mg kg^−1^, followed by raw cabbage samples with 1593.50 mg kg^−1^. The application of culinary procedures resulted in a significant decrease in nitrate concentrations. Such a reduction is a result of the good solubility of nitrates in water. According to solubility curves, nitrates dissolve well in water, and their solubility increases significantly with increasing temperature [32]. The decrease in nitrate concentration was most pronounced in spinach samples. Of the total nitrate concentration determined in the raw spinach sample, after soaking in water, cooking in water, and a combination of soaking and cooking, the nitrate concentration was reduced by 38.83, 81.64, and 86.90%, respectively. 

In white cabbage samples, the reduction was lower, 28.55, 54.47, and 62.30%, after soaking, cooking, and a combination of soaking and cooking. The weakest influence of culinary procedures was recorded for Swiss chard samples. After soaking, cooking, and a combination of soaking and cooking, the reduction in nitrate concentration was 15.35, 35.12, and 46.91%, respectively.

It is important to note that the nitrate content reduction in the Swiss chard samples was much less than expected. None of the Swiss chard samples showed a total reduction in nitrate levels, regardless of the method of culinary processing. After the most intensive processing procedures (immersion and cooking) the residual nitrate concentrations were still high (mean 1253.5 mg kg^−1^; range 147.8–3018.5 mg kg^−1^). According to Prasad, cooking reduces nitrates in different types of vegetables by an average of 47–59% [24], while these values range from 4.3 to 15.5% according to Salehzadeh [33].

In the EFSA’s Scientific opinion on possible public health risks for infants and young children from the presence of nitrates in leafy vegetables, there is no data for Swiss chard, although it is a very commonly consumed green vegetable, especially in the countries of the Mediterranean region [20]. An additional problem is that even in Regulation 915/2023, on maximum levels for certain contaminants in food, there are no maximum permissible limits for nitrates for Swiss chard. Therefore, there are no legal options for enforcing official control of these vegetables when they are on the market [30]. Recognizing this issue, several studies have been carried out with findings comparable to our results [29]. Therefore, we dare to emphasize that the results of our study are extremely worrying, especially if we apply them to children, e, especially infants, who are introduced to vegetables during the first year of life. Such children, due to their low weight, higher metabolic rate, and immature digestive system, are sensitive to the harmful effects of nitrates, which can cause methemoglobinemia [34]. Based on ADI values (0–3.7 mg kg^−1^), estimates of nitrate intake for various populations (from a toddler weighing 10 kg to an adult weighing 50 kg) are displayed in Figure 5. According to the calculation obtained and the comparison with the average and maximum values of nitrate concentrations in all analyzed chard samples, it was observed that children weighing less than 30 kg will certainly consume more than the ADI value for nitrates. Children weighing 10 kg or less will exceed the ADI value many times over. When the samples with the highest nitrate amounts are compared, all people below 80 kg will surpass the ADI limit after consuming just 100 g of chard.

In order to prevent chronic diseases, the WHO advises consuming more fruits and vegetables on a daily basis, approximately 400 g [35,36]. An increasing number of studies explain how greenhouse production and overuse of nitrogen-based fertilizers lead to an increase in nitrate and nitrite content in vegetables [37]. Human exposure to nitrates is mainly through the consumption of vegetables [38]; therefore, it is of great importance to reduce the concentration of nitrates and nitrites in it before consumption.

Compared to the Swiss chard, nitrate values found in spinach and white cabbage were lower. After cooking or a combination of soaking and cooking, at mean values, there was no concern for children weighing 10 kg to reach nitrate values higher than the ADI value. However, at the highest concentrations, these values would be exceeded.

Compared to similar studies, nitrate concentrations in white cabbage samples in our study were higher. In a particular region of Poland, white cabbage had a mean nitrate concentration of 99.8 + 21.05 mg kg^−1^ and nitrite of 0.7 + 0.06 mg kg^−1^, according to Czech results [39]. Raczuk reported that the same concentrations were 436 mg/kg and 0.88 mg kg^−1^, respectively [39]. Differences in results may be due to different growing conditions and soil types [40].

Analyzing the results it is evident that after the most intensive processing (soaking and coking) Swiss chard has a far smaller reduction (46.91%) than spinach and cabbage with 86.9% and 62.3% respectively.

According to EFSA literature, the reduction of spinach by cooking is −53%, and by cooking and washing −61% [23]. Salehzadeh described a decrease in nitrate concentrations of various types of vegetables including spinach and cabbage, but not Swiss chard, and the reduction of nitrates by cooking was 4.3–15.5% [33].

Nitrite concentrations in raw samples were significantly lower compared to nitrate concentrations. The prevalence was also significantly lower, as they were found in 33.7% of all analyzed samples, with the highest number of positive samples in spinach (15 samples), followed by Swiss chard and cabbage, 10 and 6 samples, respectively. The concentration of nitrite in all samples was significantly reduced by the cooking process. In white cabbage samples, nitrites were completely removed by the cooking and combination of soaking and cooking. In Swiss chard samples, after soaking, cooking, and a combination of soaking and cooking, the reduction in nitrite concentration was 43.39; 63.91 and 82.59%, respectively. Of the total concentration of nitrites, determined in a sample of raw spinach, after soaking in water, cooking in water and a combination of soaking and cooking, the concentration of nitrites was reduced by 39.15; 66.83 and 74.95%, respectively. According to the ADI values for nitrites (0–0.07 mg/kg of b.w.) [31], children with a body weight of 10 kg after consuming 100 g of chard or spinach with average values of nitrite concentration would exceed the ADI value. After consuming the same products with maximum nitrite content, those with significantly higher body weights would also exceed the ADI value. For white cabbage, nitrate concentration values were significantly lower and the probability of exceeding the ADI value was negligible.

Nitrates and nitrites are found in varying amounts in different kinds of vegetables. The results of the study by Sorour et al., for spinach samples, indicated higher concentrations of nitrate and nitrite compared to our results [41]. At the same time, in Swiss chard samples, nitrate concentrations were lower. Similar to our research, in spinach the reduction of nitrite by cooking was 74.1%, but in cabbage, this percentage was lower than in our research, more precisely it was 35.1% [41]. 

According to Wu and Liu, nitrite content in raw spinach was 17.1 mg kg^−1^ and in cabbage 5.7 mg kg^−1^. The nitrate reduction in spinach by cooking was 25%, while in cabbage, the nitrate reduction was significantly lower [12]. Compared to our study, the results of the Iammarino study are significantly higher, both for nitrates and nitrites [29]. 

Nitrates and nitrites in vegetables hyperaccumulate as a result of overuse of chemical fertilizers [40]. Due to a number of environmental factors, dietary consumption of nitrates and nitrites can differ greatly between locations. These elements include soil quality, local climate, agricultural practices, particular production methods, and current food production laws. These variances may result in notable disparities in the quantity of these substances present in dietary sources across various regions [42].

Depending on which nitroso molecule is produced in the GI tract, dietary nitrates, and nitrites may have positive and negative effects in the field of cancer biology. Through the enterosalivary route, nitrate-rich vegetables constitute a significant source of nitrosating precursors in the stomach. Gastric acidity has a significant impact on the conversion of salivary nitrites into either carcinogenic N-nitrosation or beneficial S-nitrosation, with less RN-NO and more RS-NO at lower gastric pH. The stomach may serve as a staging area for the development of NOCs due to elevated gastric pH [1].

Due to the high nitrate content in the analyzed Swiss chard samples, it is difficult to predict the possible effects of nitrates consumed by infants and young children due to their low body weight and immature digestive systems. Pregnant women and their unborn children are another risk category. The physiological state of pregnancy involves a significant demand for oxygen. The level of oxidative stress is likely to rise as a result of the increased intake and utilization of oxygen [43]. Additionally, exposure to nitrates depletes antioxidant reserves and causes oxidative stress. Because oxidative stress peaks during the 30th week of pregnancy, pregnant women may be more vulnerable to nitrites or nitrates inducing clinical methemoglobinemia [43].

Leafy vegetables, in addition to nitrates and nitrites that can synthesize carcinogenic nitrosamines, also contain antioxidants that can block this synthesis by reducing nitrites to nitric oxide [2,42]. Although this study does not include the analysis of antioxidants in vegetables, we dare to assume that the level of antioxidants during culinary procedures is not reduced (this hypothesis is supported by article [12], and that the antioxidant/in vivo nitrite ratio (A/N) is increased compared to the raw sample. This was especially observed in spinach, where the combination of soaking and cooking resulted in the highest reduction, with a nitrate concentration ratio factor of 10.73. Therefore, the potential health risk of nitrite was reduced. The findings of this study provide useful information for dietary recommendations, especially for vulnerable groups. It is strongly recommended to use combined culinary methods to reduce nitrate content to ensure food safety.

With regard to the reduction of nitrates during culinary procedures, it is possible to choose the consumption of different types and ways of processing green vegetables, related to the benefits and risks of nitrates and nitrites.

## 4. Materials and Methods

### 4.1. Samples 

This study examined the impact of culinary procedures on the levels of nitrates and nitrites in three varieties of green vegetables: Swiss chard (*Beta vulgaris* L.), spinach (*Spinacia oleracea*), and white cabbage (*Brassica oleracea*), commonly included in the Mediterranean diet. A total of 92 vegetable samples were tested, including 34 Swiss chard, 32 spinach, and 26 white cabbage. The samples were collected from local shops and markets in Split-Dalmatia County.

### 4.2. Sample Preparation

Approximately 1 kg of each vegetable was sampled. The damaged parts of the leaves were removed, and the samples were briefly washed under a stream of water to remove any dirt and soil that could affect the results. Samples were then divided into four equal parts that have been processed in different ways:

- ***Raw sample*** without culinary processing,
- ***Soaking***: vegetables were soaked in deionized water for 15 min at room temperature and drained in a colander
- ***Boiling*:** samples were boiled in deionized water at 100 °C for 10 min and drained.
- ***Combined soaking and boiling***: Vegetables were soaked as described above, followed by boiling.

Deionized water was used for sample preparation in order to avoid interference with chlorides from tap water [44]. Processed samples were homogenized with knife mill Grindomix GM 200 (Retsch, Haan, Germany), and 5 g (±0.01 g) of the sample was diluted with 100 mL of MeOH:H_2_O (1:1) solution. The extraction process was carried out in an ultrasonic bath for 15 min, and the resulting solution was filtered through a membrane filter, pore size 0.45 μm [45]. Qualitative and quantitative analysis of nitrates and nitrites was performed using HPLC-DAD within 24 h. Until the analyses, prepared samples were kept at a temperature of 5 °C.

### 4.3. Chemicals 

Sodium nitrate and sodium nitrite standards of high purity (>99%) were purchased from Sigma-Aldrich (Saint Louis, MO, USA) while methanol (HPLC grade) and octylamine were from Merck, Darmstadt, Germany. Deionized water (0.05 μS cm^−1^) was produced using an Omnia Pure Ultrapure water system (Stakpure GmbH, Niederahr, Germany).

### 4.4. Methods

The quantification and qualification of nitrates and nitrites in vegetables were conducted using an Agilent 1260 HPLC system (Agilent Technologies Singapore (International) Pte. Ltd., Singapore) equipped with a quaternary pump, Agilent Zorbax reversed-phase HPLC column (250 × 4.6 mm; 5 μm) and diode array detector (190–900 nm). The system is managed by OpenLab CDS Chemstation Software (Agilent, rev. C.01.05 (35)). The flow rate in the system was 1.1 mL/min and an injection volume of 10 μL. The maximum wavelength was 210 nm. The mobile phase used for HPLC-DAD analysis consisted of 0.01 M octylamine (1.29 g of octylamine in 900 mL of deionized water, adjusting the pH to 7 with 10% phosphoric acid, and filling up to 1 L) and methanol (90:10, *v*/*v*, isocratic) [45,46].

Stock standard solutions of sodium nitrate and sodium nitrite were prepared at a concentration of 1000 μg/mL by dissolving in deionized water (0.05 μS cm^−1^). Working standards were prepared daily by diluting the stock solutions with deionized water. Calibration curves with correlation coefficients > 0.999 for both analytes were established from the working standards. The limit of quantitation (LOQ) was determined to be 1.0 mg/kg for both, nitrate and nitrite and the recoveries of both analytes were >95.0%. All samples were analyzed in duplicate, ensuring a relative standard deviation (RSD) of less than 5%.

### 4.5. Statistical Analysis

The Kolmogorov–Smirnov test was used for normality checking. Due to the normal distribution of the data, continuous variables are presented with the mean and standard deviation. Groups were compared using the parametric One-Way Anova test [46]. Correlations between changes in the culinary processing factors were determined using the Pearson correlation coefficient. *p*-values of less than 0.05 were considered statistically significant [47]. The cluster graph was created using Microsoft Excel for Mac version 16.47.

## 5. Conclusions

This study provides valuable insights into the impact of different culinary processes on nitrate concentrations in leafy vegetables such as Swiss chard, spinach, and white cabbage. The results show that soaking, cooking, and a combination of these methods significantly reduced nitrate content in all samples analyzed, with the combined method showing the greatest effect. However, the reduction efficiency is not the same for all types of vegetables, with the lowest in Swiss chard samples. 

These findings are crucial for the development of dietary guidelines aimed at reducing nitrate intake, especially for vulnerable groups such as infants and young children.

The results obtained in this study, mean values and ranges, are higher than the results given in EFSA reports, but supported by other similar studies carried out recently. This suggests that a reassessment of dietary exposure to nitrates and nitrites is required particularly for children, as current amounts are significantly higher compared to the EFSA findings from 2008 and 2010.

We also emphasize that it is necessary to include a broad variety of vegetables in order to obtain relevant data on dietary exposure. 

These results may contribute to amending contamination regulations and including Swiss chard on the list of vegetables with prescribed maximum nitrate levels, given that in several studies, including this one, high levels of nitrate have been found.

## Figures and Tables

**Table 1:** Descriptive statistics concentration of nitrate in Swiss chard, spinach, and white cabbage vegetable (*n* = 92) depending on culinary processing.

Sample (*n*)	Type of Culinary Processing	Min–Max Range (mg kg^−1^)	Mean ± SD (mg kg^−1^)	One-Way ANOVA Test *p*-Value
Swiss chard (34)	raw	418.6–4723.0	2361.3 ± 1006.6	*p* < 0.001
	soaked in water	243.2–4188.9	1998.8 ± 897.6	
	boiled in water	221.8–3443.0	1532.0 ± 643.1	
	soaked and boiled	147.8–3018.5	1253.5 ± 597.3	
Spinach (32)	raw	<LOQ–3528.7	1384.5 ± 981.8	*p* < 0.001
	soaked in water	<LOQ–2527.0	846.9 ± 650.3	
	boiled in water	<LOQ–615.7	254.2 ± 182.3	
	soaked and boiled	<LOQ–534.8	181.4 ± 142.8	
White Cabbage (26)	raw	<LOQ–1593.5	690.2 ± 416.7	*p* < 0.001
	soaked in water	<LOQ–1047.3	493.1 ± 273.9	
	boiled in water	<LOQ–805.9	314.3 ± 173.9	
	soaked and boiled	<LOQ–772.7	260.2 ± 167.4	

**Table 2:** Nitrate concentration ratio factors in Swiss chard, spinach, and white cabbage depending on culinary processing.

Sample *n*	Type of Culinary Pro-Cessing	Concentration Ratio Factor Mean ± SD	One-Way ANOVA Test *p*-Value
Swiss chard 34	raw/soaked in water	1.26 ± 0.32	*p* < 0.001
	raw/boiled in water	1.61 ± 0.46	
	raw/soaked and boiled	2.05 ± 0.67	
Spinach 32	raw/soaked in water	1.69 ± 0.88	*p* < 0.001
	raw/boiled in water	5.96 ± 3.53	
	raw/soaked and boiled	10.73 ± 12.56	
White Cabbage 26	raw/soaked in water	1.43 ± 0.40	*p* < 0.001
	raw/boiled in water	2.29 ± 0.74	
	raw/soaked and boiled	2.89 ± 0.99	

## Data Availability

The data presented in this study are available upon request from the corresponding author.

## Abbreviations

GI	gastrointestinal tract
(NOCs)	N-nitroso compounds
CYP	cytochrome P450 enzymes
ADI	acceptable daily intake
EFSA	European Food Safety Authority
LOQ	Limit of quantitation
HPLC	High Pressure Liquid Chromatography
